# Phase 2a Randomized Placebo-Controlled Human Challenge Trial of the Respiratory Syncytial Virus L-Protein Inhibitor S-337395 for Respiratory Syncytial Virus Infection

**DOI:** 10.1093/cid/ciag367

**Published:** 2026-06-15

**Authors:** Stuart Byrne, Takamichi Baba, Nao Kawaguchi, Atsuko Yamamoto, Yoshiyuki Nakano, Caroline Dorrepaal, Brandon Londt, Alex Mann, Nikolay Veselinski, Takeki Uehara

**Affiliations:** Drug Development and Regulatory Science Division, Shionogi & Co., Ltd., Osaka, Japan; Drug Development and Regulatory Science Division, Shionogi & Co., Ltd., Osaka, Japan; Drug Development and Regulatory Science Division, Shionogi & Co., Ltd., Osaka, Japan; Laboratory for Drug Discovery and Disease Research, Shionogi & Co., Ltd., Osaka, Japan; Laboratory for Drug Discovery and Disease Research, Shionogi & Co., Ltd., Osaka, Japan; Medical Science Department, Shionogi B.V., London, England, United Kingdom; Clinical Sciences Department, hVIVO Services Limited, London, England, United Kingdom; Clinical Sciences Department, hVIVO Services Limited, London, England, United Kingdom; hSite Medical Operations, hVIVO Services Limited, London, England, United Kingdom; Drug Development and Regulatory Science Division, Shionogi & Co., Ltd., Osaka, Japan

**Keywords:** antiviral, human challenge, L-protein inhibitor, respiratory syncytial virus, S-337395

## Abstract

**Background:**

S-337395 is a novel inhibitor of the respiratory syncytial virus (RSV) L-protein, and a potential oral antiviral against RSV. This phase 2a, randomized, double-blind, placebo-controlled, single-center, proof-of-concept trial conducted in the United Kingdom (April-October 2024) evaluated the efficacy, safety, and dose-response relationship of S-337395 against RSV infection in a human challenge model.

**Methods:**

Healthy adults aged 18–55 years meeting predefined eligibility criteria were inoculated with RSV-A Memphis 37b on Day 0. Nasal washes were collected for quantitative reverse-transcriptase polymerase chain reaction (qRT-PCR) assessment twice daily on Days 2–12 and once on Day 13. Participants were randomized 5:13:13:13:13 to receive S-337395 (1, 10, 30, or 300 mg) or placebo, once daily for 5 days upon evidence of infection. Efficacy analyses were conducted in participants with confirmed RSV infection in intent-to-treat infected (ITT-I) set. Safety was assessed in all randomized participants. Dose-response was evaluated across a wide dose range (1–300 mg).

**Results:**

Of 114 randomized participants, 60 comprised the ITT-I set. Covariate-adjusted mean viral load area under the curve (VL-AUC) was significantly lower in the 30- and 300-mg groups by qRT-PCR (64.87% and 88.94% reductions, respectively, both *P* < .05) and by viral culture assay (72.32% and 86.17% reductions, respectively, both *P* < .05). Total symptom score AUC was 78.15% lower in the 300-mg group (*P* < .05). S-337395 was well-tolerated without any safety concerns.

**Conclusions:**

S-337395 significantly reduced viral load, viral titer, and symptoms, and was well-tolerated in this human challenge study.

**Clinical Trial Registration:** ISRCTN24865912.

## Teaser

- This phase 2a, randomized, placebo-controlled clinical trial was conducted to evaluate the efficacy, safety, and dose-response relationship of an L-protein inhibitor (S-337395) in RSV-challenged healthy participants.
- S-337395 showed significant reduction in viral load and was well-tolerated, with no safety concerns observed.

Respiratory syncytial virus (RSV) is a leading cause of respiratory diseases globally [1]. Although RSV infections occur across all age groups, a higher burden is reported among infants aged <6 months, adults with comorbidities including organ transplantation, immunocompromised state, diabetes, congestive heart failure, or obstructive pulmonary disease, and adults aged ≥65 years [1–4]. The incidence of RSV-associated lower respiratory tract infections (LRTIs) among infants aged <6 months was 96.3 per 1000 children/year as of 2024, representing ∼20% of all infections and ∼40% of all RSV-related hospitalizations [2]. As of 2019, an estimated 33.0 million RSV-associated acute LRTIs occurred globally among children aged <5 years, including 3.6 million hospitalizations and 26 300 in-hospital deaths [5]. A recent systematic review reported ∼465 873 RSV-associated hospitalizations and 33 231 in-hospital deaths in 2019 among older adults (≥60 years) in high-income countries [3].

The clinical presentation of RSV ranges from a mild cold to serious LRTIs, such as bronchiolitis in infants, and pneumonia, cardiopulmonary complications, intensive care admission, and mortality in infants and high-risk adults [2,6,7]. In children aged <5 years, RSV was noted as a predominant cause of pneumonia requiring hospital admission, accounting for 31.1% of the etiological distribution [8]. The therapeutic landscape of RSV is dominated by several promising prophylactic strategies [2]. Monoclonal antibodies (mAbs), including palivizumab, nirsevimab, and clesrovimab, are approved for pediatric populations; however, potential infection with mAb-resistant variants remains a concern [9–13]. Protein subunit vaccines such as Arexvy (GlaxoSmithKline) and mRESVIA (Moderna) for older adults, and Abrysvo (Pfizer) for older adults and infants (through maternal immunization) were recently approved. Currently, ribavirin remains the only antiviral approved solely for children undergoing RSV treatment; however, its efficacy is limited and safety concerns exist, and it is used in practice only for high-risk patients [14–16]. Despite numerous attempts to develop antiviral therapeutics targeting RSV through various mechanisms, treatments are still under investigation or limited by low efficacy and high safety concerns. Effective RSV treatment remains a major unmet need, as breakthrough infections and vaccine hesitancy contribute to RSV burden in vulnerable children and adults.

RSV is a negative-sense, single-stranded RNA virus that depends on its RNA-dependent RNA polymerase (RdRp) for transcription and synthesis of positive-sense intermediates during replication [17]. S-337395, a small-molecule oral antiviral RSV RdRp L-protein inhibitor discovered by Shionogi & Co., Ltd. and UBE Corporation, is under development for treating RSV infection. Unlike F-protein inhibitors, which only prevent viral entry into host cells, RdRp inhibitors block viral propagation in already infected cells [18]. As S-337395 achieves postentry viral inhibition, it may offer a wider therapeutic window. In vitro studies (internal data) showed comparable inhibitory effects of S-337395 against Memphis 37b (M37b) and RSV A-2 on plaque formation induced by virus replication in HEp-2 cells, with 50% effective concentrations (EC_50_) of 1.59 and 1.49 nM, respectively. In an initial phase 1 study of S-337395, single doses of 1–1200 mg and multiple doses of 200 mg and 600 mg once daily for 7 days were administered to healthy adults [19], with no safety concerns reported. In this phase 2a study, we evaluated the proof-of-concept antiviral effect, safety, and dose-response relationship of S-337395 in healthy participants challenged with RSV.

## METHODS

### Trial Design

This phase 2a, double-blind, randomized, placebo-controlled, single-center trial was conducted between April 2024 and October 2024 in the United Kingdom. Over the study duration of ∼6 months, 114 participants were enrolled across 5 dosing cohorts, with several sequential phases (Supplementary Figure 1). This study design was similar to that of previously reported RSV antiviral candidates [20–22].

### Participants

Key eligibility criteria included healthy adults aged 18–55 years, with body weight ≥50 kg and body mass index ≥18 and ≤35 kg/m^2^, serosuitable (serum titers of pre-existing RSV-A M37b–specific antibodies ≤468) for infection with RSV-A M37b. Detailed selection criteria are provided in the Supplementary Appendix.

Efficacy analysis was conducted in the intent-to-treat infected (ITT-I) and intent-to-treat infected predose (ITT-A) analysis sets. ITT-I included all randomized participants who received the challenge agent and ≥1 dose of S-337395 or placebo and had laboratory-confirmed RSV infection. The ITT-A analysis set comprised ITT-I participants with at least the first detectable quantitative reverse-transcriptase polymerase chain reaction (qRT-PCR) assessment defining RSV infection before the first dose of S-337395 or placebo (Supplementary Figure 2). The ITT-A set was considered a population closer to clinical practice—those who would receive antiviral treatment after RSV diagnosis. Efficacy analysis in the ITT-A set was prespecified and performed to reduce bias from ITT-I participants who received postexposure prophylactic treatment before testing positive for viral onset (intent-to-treat infected postdose [ITT-B]). Safety analysis was conducted in all participants who received ≥1 dose of S-337395 or placebo regardless of RSV infection status; pharmacokinetic analysis was conducted in the pharmacokinetic analysis set. The analysis sets are described in the Supplementary Appendix.

### Procedures

S-337395 and placebo were provided in powder-in-bottle form (Shionogi B.V. via a third party), reconstituted with water on site before administration, and administered orally once daily for 5 days. On Day 0, participants received a single dose of RSV-A M37b at a titer of approximately 4 log_10_ plaque-forming units (PFU), predetermined via an infectivity assay. Participants were tested for RSV PCR positivity in nasal washes twice daily, from Day 2 postinoculation up to and including Day 5 morning. Rapid testing for viral detection to trigger treatment was conducted using qualitative integrative cycler polymerase chain reaction (qicPCR) with the Simplexa™ Real-Time PCR (LIAISON MDX, Diasorin). Once RSV was detected by the qicPCR assay, participants were randomized to receive S-337395 (1, 10, 30, or 300 mg) or placebo, initiated approximately 12 hours after the first positive RSV detection and continued once daily for 5 days. All participants who had not been randomized by Day 5 evening were then randomized, and treatment was initiated on Day 5 evening, once daily for 5 days. A subtherapeutic 1-mg dose was included to evaluate the dose-response relationship.

All challenged participants were randomized 5:13:13:13:13 to groups A-E (1-mg S-337395, 10-mg S-337395, 30-mg S-337395, 300-mg S-337395, and placebo, respectively), using a 4-digit computer-generated code. For assignment of laboratory-confirmed RSV infection to analysis sets (ITT-I, ITT-A, and ITT-B), and for efficacy and dose-response evaluation, quantitative RSV assessments of nasal wash samples were conducted twice daily from Day 2 morning to Day 13 morning. Viral load analysis, including qRT-PCR and RSV plaque assays, was performed by hVIVO Services Limited. Participants’ clinical symptoms were assessed thrice daily using a symptom diary card with a 13-item questionnaire on a graded scale of 0;3, with higher scores indicating greater severity. Safety and pharmacokinetics were assessed throughout the trial. For safety assessment, adverse events (AEs) were coded using the Medical Dictionary for Regulatory Activities (MedDRA; version 27.0). Blood samples were collected to measure plasma concentrations and calculate pharmacokinetic parameters of S-337395. Further details are provided in the Supplementary Appendix.

### Endpoints

The primary endpoint was efficacy, determined as the RSV viral load area under the curve (VL-AUC), measured by qRT-PCR of nasal wash samples from initial administration of S-337395 or placebo up to planned discharge (Day 13). Secondary efficacy endpoints included viral load measured by a cell-based viral culture infectivity assay, time to cessation of viral detection by qRT-PCR and viral culture assays, and AUC measurement for total symptom score (TSS-AUC). Safety endpoints included the incidence of AEs. Exploratory endpoints included the pharmacokinetics of S-337395 assessed throughout, and more frequently on Days 1 and 5, including maximum plasma concentration (*C*_max_), plasma concentration 24 hours postdose and before subsequent dose (*C*_trough_), time to *C*_max_ (*T*_max_), AUC for the concentration-time curve over the dosing interval (AUC_0-τ_), and terminal elimination half-life (*t*_1/2,z_).

### Sample Size Estimation

Assuming 90% relative reduction in VL-AUC versus placebo for each S-337395 group, 62% infection rate, and a coefficient of variation (CV) of 0.66 for VL-AUC in the placebo group, a sample size of 104 evaluable participants was calculated to result in 64 infected participants (16 per group) and was deemed suitable for each main comparison (10- to 300-mg groups vs placebo), providing 96.2% power for superiority to placebo and 80.4% probability of achieving >70% relative reduction in VL-AUC versus placebo. A sample size of 10 participants (6 infected) was estimated for the 1-mg group, assuming 20%–50% relative reduction and 5.7%–26.3% probability of achieving >70% comparative reduction.

### Statistical Analysis

Continuous variables were summarized as mean, standard deviation (SD), median, minimum, and maximum, and categorical variables as frequencies and percentages. Two-sided 95% confidence intervals (CIs) were calculated using the Clopper–Pearson method. VL-AUC was calculated using log_10_-transformed PCR data, and between-group pairwise comparisons of placebo versus each S-337395 group (10- to 300-mg) were conducted using an analysis of covariance model, with each group as the main effect, and the baseline qRT-PCR viral load (last before S-337395 or placebo administration) as a covariate. Pairwise comparisons between each active group and placebo were reported as covariate-adjusted mean differences, 95% CIs, and *P* values. Statistical significance was considered at a two-sided *P* value <.05. Kaplan–Meier curves were used for time-to-event analyses, and log-rank tests were performed to compare each active drug with the placebo group. Data imputations were limited to some graphs.

## RESULTS

### Demographic Details

Of 114 enrolled participants, 10 were randomized to the 1-mg group and 26 each to the 10-mg, 30-mg, 300-mg, and placebo groups (Supplementary Figure 2). Only 1 participant (30-mg group) discontinued due to an unforeseen family emergency. In the ITT-I population (n = 60), the proportion of men was higher (71.7%; Table 1). The mean ± SD age of participants was 27.17 ± 6.78 years and was generally comparable across all groups. Baseline characteristics of the ITT-A and ITT-I populations were comparable (Supplementary Table 1).

**Table 1.** Demographic Characteristics (ITT-I Analysis Set)

Description	Placebo	S-337395				All
		1 mg	10 mg	30 mg	300 mg	
	(n = 21)	(n = 6)	(n = 13)	(n = 11)	(n = 9)	(N = 60)
Sex, n (%)						
Female	5 (23.8)	2 (33.3)	7 (53.8)	2 (18.2)	1 (11.1)	17 (28.3)
Male	16 (76.2)	4 (66.7)	6 (46.2)	9 (81.8)	8 (88.9)	43 (71.7)
Age, years						
Mean ± *SD*	25.24 ± 5.73	30.17 ± 9.39	26.69 ± 5.04	30.45 ± 7.89	26.33 ± 7.33	27.17 ± 6.78
Median (min, max)	24.00 (18.0, 40.0)	27.00 (19.0, 43.0)	25.00 (19.0, 38.0)	28.00 (21.0, 49.0)	25.00 (20.0, 44.0)	25.50 (18.0, 49.0)
Race, n (%)						
Asian	2 (9.5)	0 (0.0)	1 (7.7)	2 (18.2)	0 (0.0)	5 (8.3)
Black or African American	0 (0.0)	0 (0.0)	1 (7.7)	0 (0.0)	0 (0.0)	1 (1.7)
White	15 (71.4)	6 (100.0)	10 (76.9)	8 (72.7)	8 (88.9)	47 (78.3)
Other	1 (4.8)	0 (0.0)	1 (7.7)	0 (0.0)	1 (11.1)	3 (5.0)
Multiple	3 (14.3)	0 (0.0)	0 (0.0)	1 (9.1)	0 (0.0)	4 (6.7)
Ethnicity, n (%)						
Hispanic or Latino	0 (0.0)	0 (0.0)	0 (0.0)	0 (0.0)	0 (0.0)	0 (0.0)
Not Hispanic or Latino	21 (100.0)	6 (100.0)	13 (100.0)	11 (100.0)	9 (100.0)	60 (100.0)

Abbreviations: ITT-I, intent-to-treat infected; max, maximum; min, minimum; *SD*, standard deviation.

### Efficacy Analysis

#### Viral Load Analysis by qRT-PCR

In the ITT-I set, covariate-adjusted mean (95% CI) VL-AUC was lowest in the 300-mg group (77.25 [−140.14, 294.64]) and significantly lower versus placebo (698.73 [557.56, 839.90]), with a relative difference of −88.94% (*P* < .05) (Figure 1*A* and Table 2). A statistically significant decrease in covariate-adjusted mean VL-AUC was observed in the 30-mg group (245.48 [53.24, 437.73]; *P* < .05), with a relative difference of −64.87%. The mean differences were not statistically significant for the 1- and 10-mg groups. A dose-dependent reduction in VL-AUC was observed in the 10- to 300-mg range. The ITT-A analysis set showed similar results (Supplementary Table 2).

**Figure 1.**
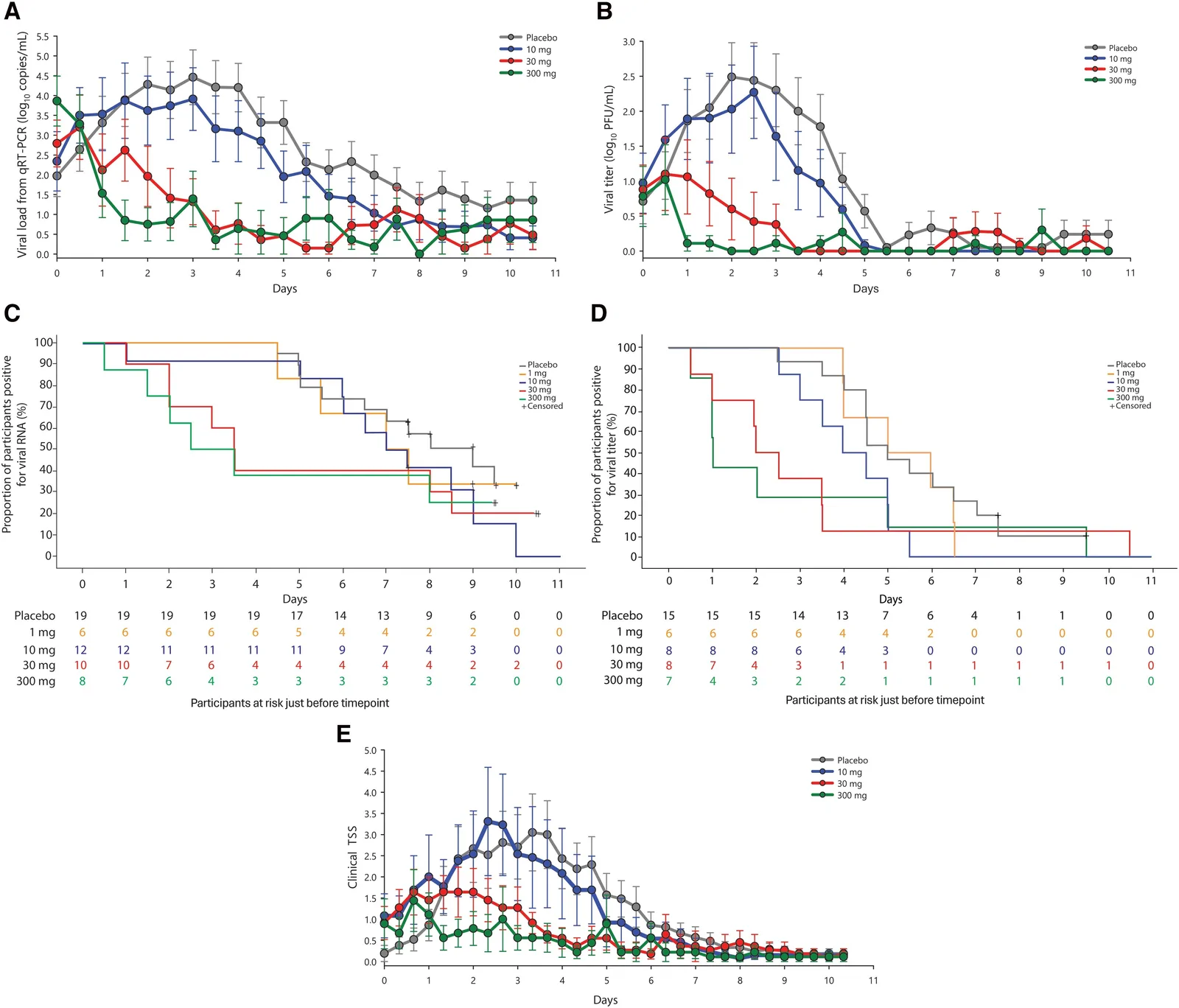
Efficacy analysis: (*A*) Viral load analysis by qRT-PCR, (*B*) viral load analysis by viral culture assay, (*C*) dose-response with respect to time, demonstrating mean time from baseline to cessation of virus detection by qRT-PCR (ITT-I analysis set), (*D*) dose-response with respect to time, demonstrating mean time from baseline to cessation of virus detection by viral culture assay (ITT-I analysis set), and (*E*) analysis of clinical symptom resolution (ITT-I analysis set). The primary and secondary efficacy endpoints were assessed in the ITT-I analysis set, with viral load and symptom score represented as mean ± SE. Data after discharge were imputed using the last-observation-carried-forward method. Abbreviations: ITT-I, intent-to-treat infected; PFU, plaque-forming unit; qRT-PCR, quantitative reverse-transcriptase polymerase chain reaction; SE, standard error; TSS, total symptom score.

**Table 2.** Efficacy Analysis (ITT-I Analysis Set)

Description	Placebo	S-337395			
		1 mg	10 mg	30 mg	300 mg
	(n = 21)	(n = 6)	(n = 13)	(n = 11)	(n = 9)
VL-AUC determined by qRT-PCR (hours × log_10_ copies/mL)^a^					
Mean ± *SD*	629.42 ± 468.48	771.27 ± 449.97	516.74 ± 444.56	263.70 ± 258.64	208.94 ± 195.22
Covariate-adjusted mean (95% CI)	698.73 (557.56, 839.90)	696.20 (434.71, 957.68)	546.01 (368.97, 723.05)	245.48 (53.24, 437.73)	77.25 (−140.14, 294.64)
Difference in covariate-adjusted mean^b^ (95% CI)	;	−2.53 (−301.85, 296.78)	−152.72 (−378.05, 72.61)	−453.25 (−692.41, −214.08)	−621.48 (−885.00, −357.96)
*P* value^c^	;	0.9865	0.1798	0.0004	<0.0001
Relative difference (%)^d^	;	−0.36	−21.86	−64.87	−88.94
VL-AUC determined by viral culture (hours × log_10_ PFU/mL)^a^					
Mean ± *SD*	227.38 ± 201.58	279.42 ± 273.29	175.04 ± 199.18	70.47 ± 102.73	30.64 ± 30.62
Covariate-adjusted mean (95% CI)	233.92 (168.62, 299.21)	292.09 (169.93, 414.25)	162.31 (79.23, 245.38)	64.75 (−25.43, 154.93)	32.35 (−67.32, 132.01)
Difference in covariate-adjusted mean^b^(95% CI)	;	58.17 (−80.25, 196.60)	71.61 (−177.40, 34.18)	−169.17 (−280.55, −57.78)	−201.57 (−320.70, −82.43)
*P* value^c^	;	0.4032	0.1804	0.0036	0.0013
Relative difference (%)^d^	;	24.87	−30.61	−72.32	−86.17
TSS-AUC from baseline up to Day 13 morning (hours × score)^a,e^					
Mean ± *SD*	310.50 ± 385.93	286.74 ± 297.33	294.19 ± 430.60	170.03 ± 172.69	107.09 ± 146.69
Covariate-adjusted mean (95% CI)	363.66 (231.41, 495.91)	304.09 (61.66, 546.53)	244.80 (78.33, 411.27)	140.06 (−39.52, 319.63)	79.45 (−118.86, 277.76)
Difference in covariate-adjusted mean^b^ (95% CI)	;	−59.57 (−334.88, 215.75)	−118.86 (−334.59, 96.87)	−223.60 (−448.43, 1.22)	−284.21 (−524.13, −44.28)
*P* value^c^	;	0.6662	0.2742	0.0512	0.0211
Relative difference (%)^d^	;	−16.38	−32.68	−61.49	−78.15

Abbreviations: ANCOVA, analysis of covariance; CI, confidence interval; ITT-I, intent-to-treat infected; PFU, plaque-forming unit; qRT-PCR, quantitative reverse-transcriptase polymerase chain reaction; *SD*, standard deviation; TSS, total symptom score; TSS-AUC, area under the curve measurement for TSS; VL-AUC, viral load area under the curve.

^a^Baseline is the last nonmissing observation before administration of the first dose of S-337395 or placebo.

^b^Comparison of each active dose with placebo.

^c^ *P* values are from ANCOVA, with treatment group as the main effect and baseline value as covariate.

^d^Relative difference is calculated as follows: (difference in covariate-adjusted mean for each active group/covariate-adjusted mean for placebo) × 100.

^e^TSS is the sum of the following 10 symptoms: runny nose, stuffy nose, sneezing, sore throat, earache, malaise/tiredness, headache, muscle/joint ache, cough, and shortness of breath.

#### Viral Load Analysis by Viral Culture Assay

Significant reductions in covariate-adjusted mean (95% CI) VL-AUC were observed in the 30-mg (64.75 [−25.43, 154.93]) and 300-mg (32.35 [−67.32, 132.01]) groups, with respective relative differences of −72.32% and −86.17% (both *P* < .05) versus placebo (233.92 [168.62, 299.21]). With a relative difference of −30.61% in the 10-mg group, a trend toward a dose-dependent decrease in viral load was observed (Figure 1*B* and Table 2). The ITT-A analysis set showed comparable results (Supplementary Table 2).

The median time to cessation of virus detection was substantially shorter in the 30- and 300-mg groups versus placebo by qRT-PCR (3.5 and 3.0 vs 9.0 days, respectively) and by viral culture (2.3 and 1.0 vs 5.0 days, respectively; Figure 1*C* and 1*D*).

### Symptom Score Analysis

RSV clinical symptom outcomes aligned with viral load results in the ITT-I population. Covariate-adjusted mean (95% CI) TSS-AUC in the 300-mg group (79.45 [−118.86, 277.76]) was significantly lower versus placebo (363.66 [231.41, 495.91]), with a relative difference of −78.15% (*P* < .05). Differences in other groups were not statistically significant (Table 2). Mean TSS-AUC showed a rapid dose-dependent decrease (Figure 1*E*). In the ITT-A analysis set, TSS decline was slightly more pronounced versus the ITT-I population; TSS-AUC showed significantly greater reductions in both 30-mg (67.84%) and 300-mg (81.67%) groups versus placebo (Supplementary Table 2).

### Safety Analysis

Thirty-two (28.1%) participants reported 37 AEs, of which 30 occurred after the first administration of S-337395 or placebo (Table 3). All AEs were mild or moderate. No serious AEs, deaths, or AEs leading to discontinuation were reported. Treatment-emergent AEs (TEAEs) were reported in 2/26 (7.7%) participants in the placebo group. Among the S-337395-treated groups, 26/88 (29.5%) participants experienced TEAEs, with no specific trend or dose dependency. TEAEs between postdischarge and the end of the study occurred in 1/26 (3.8%) participants in the placebo group and in 15/88 (17.0%) participants across the S-337395-treated groups (Table 3). The most common TEAE was upper respiratory tract infection, reported in 3/114 (2.6%) participants. No other TEAE was reported in >1 participant within a single treatment group (Supplementary Table 3). All TEAEs resolved by study completion, except for 1 case of plantar dermatitis unrelated to the challenge agent, S-337395, or placebo, which recovered with a sequelae of dry skin. No treatment-related TEAEs were reported.

**Table 3.** Summary of AEs (Safety Analysis Set)

Description	Placebo	S-337395				All
		1 mg	10 mg	30 mg	300 mg	
	(n = 26)	(n = 10)	(n = 26)	(n = 26)	(n = 26)	(N = 114)
Any AE	2 (7.7) [2]	4 (40.0) [5]	6 (23.1) [8]	11 (42.3) [11]	9 (34.6) [11]	32 (28.1) [37]
Any CEAE	2 (7.7) [2]	4 (40.0) [5]	6 (23.1) [8]	11 (42.3) [11]	9 (34.6) [11]	32 (28.1) [37]
Any CEAE considered (at least possibly) related to the challenge virus	2 (7.7) [2]	1 (10.0) [1]	1 (3.8) [2]	2 (7.7) [2]	1 (3.8) [1]	7 (6.1) [8]
Any TEAE	2 (7.7) [2]	4 (40.0) [5]	6 (23.1) [7]	10 (38.5) [10]	6 (23.1) [6]	28 (24.6) [30]
Any TEAE considered (at least possibly) related to the challenge virus	0	0	0	0	0	0
Any AE leading to study discontinuation	0	0	0	0	0	0
Any SAE or death	0	0	0	0	0	0
Any CEAE between Day 0 and predose	0	0	1 (3.8) [1]	1 (3.8) [1]	4 (15.4) [5]	6 (5.3) [7]
Any TEAE between postdose and discharge	1 (3.8) [1]	3 (30.0) [4]	5 (19.2) [5]	2 (7.7) [2]	2 (7.7) [2]	13 (11.4) [14]
Any TEAE between post-discharge and end of study	1 (3.8) [1]	1 (10.0) [1]	2 (7.7) [2]	8 (30.8) [8]	4 (15.4) [4]	16 (14.0) [16]

Safety outcomes are presented as the number of participants (percentage) [number of events].

Abbreviations: AE, adverse event; CEAE, challenge-emergent AE; SAE, serious AE; TEAE, treatment-emergent AE.

## Pharmacokinetic Analysis

Following once-daily multiple doses of 300-mg S-337395 for 5 days, *C*_max_, *C*_trough_, and AUC_0-τ_ on Day 1 were 1610 ng/mL, 502 ng/mL, and 22 560 ng h/mL, respectively, correspondingly increasing to 2620 ng/mL, 972 ng/mL, and 40 710 ng h/mL on Day 5 (Supplementary Table 4). The mean ± *SD* plasma concentration profile of S-337395 over 8 days exhibited a less-than-dose-proportional increase (Figure 2). Geometric mean *t*_1/2,z_ on Day 5 after multiple doses was 16.1–16.9 hours across all regimens and was therefore dose-independent. *C*_max_ and AUC_0-τ_ of S-337395 exhibited moderate-to-high interindividual variability (%CV geometric mean range: 22.0%–43.9% on Day 1; 29.6%–52.8% on Day 5).

**Figure 2.**
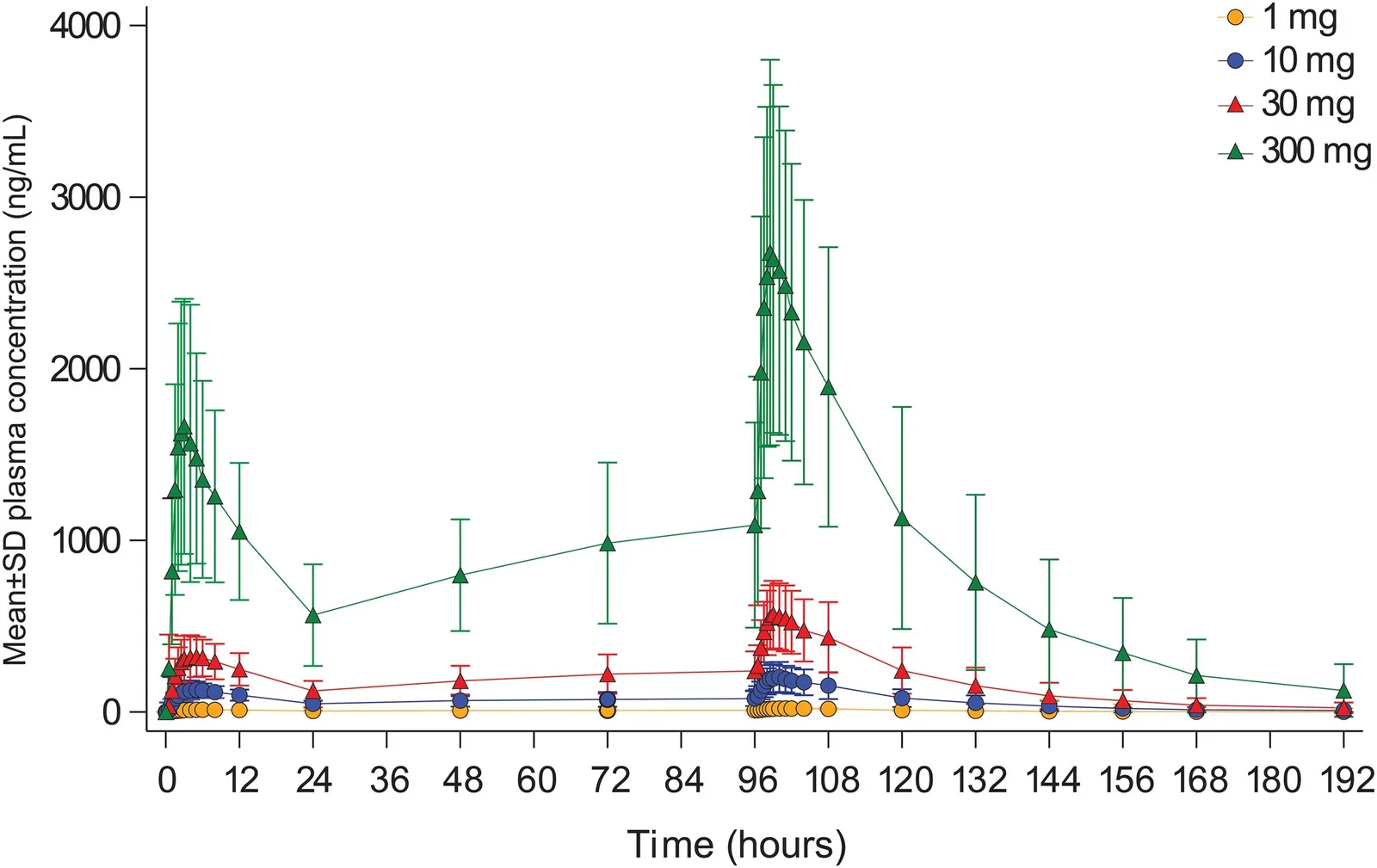
Mean ± *SD* plasma concentration profiles of S-337395 after multiple doses of S-337395 (pharmacokinetic analysis set). Mean ± *SD* is presented on a linear scale. Abbreviation: *SD*, standard deviation.

## DISCUSSION

This phase 2a study assessed the safety, pharmacokinetics, and proof-of-concept efficacy of S-337395, an RSV L-protein inhibitor, in RSV-challenged healthy adults. The antiviral effect showed a clear dose-response relationship, with evident efficacy in reducing viral load and clinical symptoms at ≥30-mg doses. Despite a 300-fold dose range, exposure increased in a less-than-dose-proportional manner, with a ∼100-fold exposure range. S-337395 was well-tolerated at all dose regimens up to 300 mg, with no safety concerns identified. These results align with the phase 1 study of S-337395 [19]—the first-in-human study among healthy Japanese and Caucasian participants reporting the safety and tolerability of S-337395, with a negligible food effect.

In this study, S-337395 administration (30 mg and 300 mg) once daily for 5 days demonstrated statistically significant covariate-adjusted relative reductions (*P* < .05) in both viral load determined by qRT-PCR (64.87% and 88.94%, respectively) and viral culture (72.32% and 86.17%, respectively) versus placebo. The corresponding median time to cessation of virus detection was also shorter, although not statistically significant. Mean TSS-AUC showed a dose-dependent decrease, with a statistically significant covariate-adjusted mean reduction of 78.15% (*P* < .05) in the 300-mg group. Thus, this study provides proof-of-concept for maximal antiviral effect with the 300-mg regimen.

To date, translation of results from human challenge studies to real-world clinical settings remains challenging. Presatovir, an oral RSV F-inhibitor, showed significantly reduced viral load and improved clinical symptoms in healthy adults with experimental RSV infection [23], but its phase 2 trial reported no significant effect on viral load in hematopoietic cell transplantation recipients with RSV infection [24].

Obeldesivir (RdRp inhibitor, Gilead) and F-inhibitors such as sisunatovir (Pfizer) and rilematovir (Janssen) are high-profile antivirals for RSV that have been discontinued in recent years. In practice, RSV symptoms often appear after the virus enters host cells, rendering F-inhibitors ineffective following viral entry and highlighting the challenges of antiviral development [25–27]. In contrast, L-inhibitors can inhibit the viral replication cycle following cell entry.

A recently developed oral L-protein inhibitor, EDP-323, successfully inhibited RSV propagation in vitro. The EDP-323 phase 1 study in healthy participants reported high tolerability up to 800 mg once daily for 7 days; mild side effects were deemed unlikely to be treatment-associated, and t_1/2,z_ was 10.8–16.6 hours [28]. The current study showed a similar safety profile and comparable t_1/2,z_ of 16.1–16.9 hours. Furthermore, a phase 1/2 trial of PC786, another RdRp inhibitor, showed significant antiviral effects; however, the study was underpowered, with the formulation reportedly not optimized for nasal delivery. Moreover, the study model did not include any subgroup mimicking natural infections in at-risk populations [29].

Our ITT-A population (used for sensitivity analysis) showed efficacy outcomes comparable to the ITT-I group, with the largest numerical reduction in viral RNA load. These findings aligned with our expectations; as the ITT-B group effectively received postexposure prophylactic treatment before the detectable onset of infection, its exclusion could minimize potential noise in the overall ITT-I analysis. Based on these results, a subsequent phase 2b trial evaluating the safety, tolerability, and efficacy of S-337395 in adults with a high risk of severe disease and a narrow window from symptom onset has been initiated [30]. The demonstration of a clear dose-response relationship is notable. The dose ranges investigated in previous challenge studies for other agents were often narrow. In this study, evaluation of a wide dose range revealed a clear dose-response relationship, providing valuable information for dose selection in subsequent studies.

Nevertheless, this study faces some limitations intrinsic to human challenge studies. Frequent PCR testing for RSV following viral challenge facilitates rapid treatment commencement, which may not be feasible in clinical settings. Furthermore, the translation of human challenge study results to the clinical setting may have additional limitations, as RSV challenge uses a single RSV-A strain and includes an immunocompetent population. In this challenge study, estimations were carried out to ensure that a sufficient sample size was included to demonstrate efficacy, albeit with a small number of planned participants. While more participants will be included in subsequent phase 2/3 studies of S-337395 to confirm efficacy within target populations, the strength of the outcomes observed in this study, despite the small number of participants, exemplifies the added value of performing a well-controlled human challenge study.

## CONCLUSIONS

S-337395 demonstrated superior efficacy in reducing both viral load and symptom scores at 30- and 300-mg doses versus placebo. Moreover, S-337395 showed a dose-dependent antiviral effect and was well-tolerated in this human challenge study. These positive results support further development of S-337395 as a potential oral treatment for RSV infection.

## Supplementary Material

ciag367_Supplementary_Data

## Supplementary Data

Supplementary materials are available at *Clinical Infectious Diseases* online. Consisting of data provided by the authors to benefit the reader, the posted materials are not copyedited and are the sole responsibility of the authors, so questions or comments should be addressed to the corresponding author.
