# Supplementary material for: Phase 2a Randomized Placebo-Controlled Human Challenge Trial of the Respiratory Syncytial Virus L-Protein Inhibitor S-337395 for Respiratory Syncytial Virus Infection
Source: Clin Infect Dis. 2026 Jun 15;83(3):e652–60. doi: 10.1093/cid/ciag367 (PMC13644856; doi:10.1093/cid/ciag367)
Supplement: Supplementary file 1 [file ciag367_supplementary_data.docx]

**SUPPLEMENTARY APPENDIX**

The authors have included this appendix to provide readers with additional information about their work.

Supplement to: Phase 2a Randomized Placebo-Controlled Human Challenge Trial of the RSV L-Protein Inhibitor S-337395 for Respiratory Syncytial Virus Infection

**Authors:** Stuart Byrne^1^, Takamichi Baba^1^, Nao Kawaguchi^1^, Atsuko Yamamoto^2^, Yoshiyuki Nakano^2^, Caroline Dorrepaal^3^, Brandon Londt^4^, Alex Mann^4^, Nikolay Veselinski^5^, Takeki Uehara^1*^

**Affiliations:**

^1^Drug Development and Regulatory Science Division, Shionogi & Co., Ltd., Osaka, Japan

^2^Laboratory for Drug Discovery and Disease Research, Shionogi & Co., Ltd., Osaka, Japan

^3^Medical Science Department, Shionogi B.V., London, England, United Kingdom

^4^Clinical Sciences Department, hVIVO Services Limited, London, England, United Kingdom

^5^hSite Medical Operations, hVIVO Services Limited, London, England, United Kingdom

**^*^Correspondence to:**

**Name:** Takeki Uehara

**Address:** Drug Development and Regulatory Science Division, Shionogi & Co., Ltd., GRAND GREEN OSAKA South Building Park Tower, 5-54 Ofuka-cho, Kita-ku, Osaka 530-0011, Japan

**Telephone number:** +81-6-6202-2161

**Fax number:** NA

**Email address:** takeki.uehara@shionogi.co.jp

**ORCID iD:** 0000-0002-5853-9426

# 1. Description of study intervention

The investigational medicinal product (IMP) administered in this study was either S-337395 or placebo. S-337395 is a respiratory syncytial virus (RSV) ribonucleic acid (RNA)-dependent RNA polymerase (RdRp) inhibitor discovered by Shionogi & Co., Ltd. and UBE Corporation, and is currently under development for the treatment of RSV infection. S-337395 inhibits RSV replication by inhibiting the RdRp activity of the L‑protein involved in the transcription or replication of the RSV genome. Briefly, the interventions were administered as follows in this study:

- S-337395 powder for oral suspension
  - Dose: 1 mg, 10 mg, 30 mg, or 300 mg once daily for 5 days
  - Mode of administration: oral
- Placebo: matching S-337395 powder for oral suspension
  - Dose: not applicable
  - Mode of administration: oral

Furthermore, a challenge agent, RSV-A Memphis 37b (M37b) virus, was used as part of the human challenge test, with a volume or titer of ⁓4 log_10_ plaque-forming units (PFUs) and intranasal administration.

The duration of the study intervention was 6 days (human viral challenge [HVC] on Day 0+5 days of IMP administration). There was a lag period between HVC on Day 0 and IMP administration; IMP administration was initiated upon confirmation of infection, but no later than the evening of Day 5.

# 2. Inclusion and exclusion criteria

***Inclusion criteria were as follows:***

- Healthy adult male and female participants aged 18–55 years (inclusive) on the day before signing the study-specific informed consent form
- Participants with total body weight ≥50 kg and body mass index ≥18 kg/m^2^ and ≤35 kg/m^2^
- Participants serosuitable for infection with the RSV-A M37b challenge virus, as determined by the neutralization antibody assay (suggesting that the participant was sensitive to RSV-A M37b virus infection, i.e., likely to be infected following inoculation with the challenge agent)
- Participants in good health with no history or current evidence of clinically significant medical conditions or abnormalities that may interfere with participant safety, as determined by medical history, physical examination (including vital signs), electrocardiogram, and routine laboratory tests, as assessed by the investigator
- Participants with a documented medical history either before entering the study or participants followed up with a medical history review with the study physician at the time of screening
- Male and female participants who agreed to consistent contraceptive use during the study intervention period and for at least 90 days after the last dose of study intervention
  - Male participants were eligible if—for the duration of the study and at least 90 days after the last dose of IMP—they refrained from donating fresh unwashed semen, either remained abstinent from heterosexual intercourse or used a male condom during intercourse, and were advised of the added benefit of their female partner using a highly effective contraceptive method. A male condom was also required during any activity that could allow ejaculate transfer
  - Female participants were eligible if they were not pregnant or breastfeeding, and were either not of childbearing potential or were of childbearing potential and agreed to use a highly effective, low user-dependent contraceptive method during the study and for at least 28 days after the last dose of IMP. The investigator assessed the risk of contraceptive failure before study initiation. Female participants of childbearing potential were required to have a negative serum pregnancy test before enrollment

***Exclusion criteria were as follows:***

***Medical history***

- Participants with a history of, or currently active, symptoms or signs suggestive of upper or lower respiratory tract infection within 4 weeks before the first study visit
- Participants with a history of any clinically significant, or currently active, cardiovascular, respiratory, dermatological, gastrointestinal, endocrinological, hematological, hepatic, immunological (including immunosuppression), metabolic, urological, renal, neurological, or psychiatric disease, or any other major disease that may interfere with completion of the study and necessary investigations
- Participants who had smoked ≥10 pack-years at any time (10 pack-years was considered equivalent to one pack of 20 cigarettes/day for 10 years)
- Female participants who had been pregnant within 6 months before study entry
- Participants with a lifetime history of anaphylaxis and/or severe allergic reaction
- Participants with significant intolerance to any food or drug within the last 12 months
- Participants with venous access deemed inadequate for the phlebotomy and cannulation demands of the study
- Participants with any significant nasal or nasopharyngeal anatomical abnormality, including a history of large nasal polyps, recent clinically significant epistaxis (major nosebleeds), or nasal or sinus surgery within 3 months before the first study visit, that could interfere with nasal assessments or viral challenge

***Prior or concomitant medication and assessments***

- Participants with vaccination within 4 weeks before the planned date of viral challenge or intention to receive any vaccination(s) before the last day of follow-up
- Participants with receipt of blood or blood products, or blood loss (including blood donations) of ≥550 mL within 3 months before the planned date of viral challenge or planned during the 3 months after the final visit
- Participants with use or anticipated use of concomitant medications (prescription and/or non-prescription), including over-the-counter drugs (if doses taken within 7 days before viral challenge exceeded the maximum permissible 24-hour dose), vitamins and dietary supplements (within 21 days before viral challenge), or herbal supplements (within 7 days before viral challenge), that could potentially interfere with the study
- Participants with receipt of any investigational drug within 3 months or five half-lives of the IMP used in the other study, whichever was longer, before the planned date of viral challenge; receipt of three or more investigational drugs in the past 12 months; prior inoculation with a virus from the same family as the challenge agent; or participation in another HVC study with a respiratory virus within the past 3 months
- Participants with a confirmed positive test for drugs of misuse or cotinine at the first study visit (with one repeat test permitted at the principal investigator’s discretion); history or presence of alcohol addiction or excessive alcohol use (>28 units/week); or excessive intake of xanthine-containing substances (eg, >5 cups of caffeinated beverages daily)
- Participants with a forced expiratory volume in 1 second (FEV1) <80%
- Participants with a positive test for human immunodeficiency virus, hepatitis B virus, or hepatitis C virus
- Participants with fever, defined as a temperature reading of ≥37.9°C on Day −2, Day −1, and/or pre-HVC on Day 0

***Other***

- Participants employed with or immediate relatives of those employed with hVIVO or the sponsor
- Participants with any medical, psychiatric, social, or occupational condition—or any other reason—that, in the investigator’s opinion, could interfere with protocol adherence, safety assessment, or the participant’s ability to comply with quarantine requirements

# 3. Definitions of the analysis sets of participants

- Primary and some secondary efficacy parameters were analyzed in the intent-to-treat infected (ITT-I) analysis set (n=60), which included participants who received RSV and ≥1 dose of IMP, with laboratory-confirmed RSV infection
- For some secondary efficacy parameters, the intent-to-treat infected pre-dose (ITT-A) analysis set (n=49) was used. It included all randomized participants who met the above criteria of the ITT-I set and had at ≥1 detectable quantitative reverse-transcriptase polymerase chain reaction (qRT-PCR) assessment defining infection before the first dose of IMP was administered
- The safety analysis set included participants who received RSV-A M37b, regardless of whether they received IMP (n=114)
- The pharmacokinetic set, used in the exploratory pharmacokinetic evaluation, included all participants with RSV challenge who received S-337395 (n=88)

# 4. Method details

## *Virological assay methods*

- Nasal wash samples were collected using 5 mL of normal saline per naris and stored for viral load analysis per the standard procedure [1]. Briefly, nasal wash samples were brought immediately to the laboratory after collection on wet ice and were processed as follows: 1) The samples were pulse-vortexed to break mucus clumps. If the clumps persisted, the samples were further homogenized by pipette; and 2) The aliquots were collected immediately and diluted 1:1 with hVIVO’s proprietary 2X stabilization transport media (STM) for plaque assay. These samples were transferred to −80ºC refrigeration for storage. Aliquots used for qRT-PCR and qualitative PCR were not diluted in STM [1].
- Qualitative integrative cycler PCR (qicPCR): To trigger treatment administration/dosing, the onset of viral shedding was evaluated using a real-time reverse transcription-polymerase chain reaction method for the rapid detection of RSV which has previously been described in detail by Kelly et al., 2015 [2]. Each 50 µL of nasal wash was mixed with 50 µL of reagent from the Simplexa™ Flu A/B and RSV reaction kit (Focus Diagnostics, Cypress, CA, USA) in a single disc wedge of a Simplexa 8-well Direct Amplification Disc (Focus Diagnostics, Cypress, CA, USA). The Direct Amplification Discs were processed and amplified within a 3M Integrated Cycler (3M, Saint Paul, MN, USA), which detected the presence of target RSV viral RNA in each sample by real-time fluorometry. Amplification plots were used to evaluate the positive or negative results for each sample. RSV infection positive interpretation was recorded using a pre-defined cycle threshold (Ct) cut-off value of Ct >0 and ≤31 as positive for RSV.
- qRT-PCR: RSV RNA was extracted from nasal wash samples using a Qiagen DSP Virus/Pathogen Midi kit (Qiagen, Hilden, Germany) on the QIASymphony SP module (Qiagen, Hilden, Germany). Each extracted RNA sample was then loaded in triplicate via the QIASymphony AS module (Qiagen, Hilden, Germany) into the QuantiFAST mastermix (Qiagen, Hilden, Germany) containing an RSV Taqman hydrolysis probe (Qiagen, Hilden, Germany) and PCR primers targeting the RSV genome (Qiagen, Hilden, Germany). The two-step quantitative RT-PCR reaction was performed using a ViiA7 thermocycler (Applied Biosystems, Waltham, MA, USA) for up to 40 cycles [1]. The quantity of viral RNA was determined by comparing Ct values to a standard calibration curve for the target-specific copy number, which was prepared from in vitro−generated nucleic acid copies of the target RSV gene sequences and run with each batch of samples. The RNA quantity mean, calculated by the ViiA7 software was then used to determine the final viral load from each sample. During the validation of assay methodology, the lower limit of quantification (LLOQ) in the assay was determined as a Ct of 32.53, which corresponded to a viral load of 3.19 log_10_ copies/mL. These validation experiments governed by repeat testing of known viral load, were performed in accordance with both the United States Food and Drug Administration and the International Conference on Harmonisation guidelines. LLOQ was defined as the Ct associated with the lowest concentration of virus at which all replicates were detected within the expected range [1]. The viral load of test samples with a Ct of ≤32.53 was determined from the standard curve. All positive qRT-PCR results with a Ct of >32.53 were reported as “detected”; however, viral load was not calculated for the other samples because it was less than the validated LLOQ of the assay.
- Plaque assay: Human epithelial type 2 (HEp-2) cells were seeded into 24-well assay plates at 5 × 10^5^ cells/mL cell density. The plates were incubated at 37ºC (±2ºC) under 5% CO_2_ overnight and used at 80%-90% confluence on the day of the assay. Nasal wash samples were added to the assay plate wells at four different dilutions (each dilution in triplicate), followed by incubation for 1 hour at 37ºC (±2˚C) under 5% CO_2_, followed by inoculation of methyl cellulose media (Sigma-Aldrich, St. Louis, MO, USA). The assay plates were incubated for 6 days at 37˚C (±2˚C) under 5% CO_2,_ fixed with 10% neutral buffered formalin fixative, stained with hematoxylin (0.06% v/v) and eosin Y (1% v/v) solutions, and then dried overnight. The presence of plaques and their count per assay plate were determined by light microscopy. For each dilution, the virus titer was calculated as the average number of plaques counted for the three replicate wells and represented as log_10_ PFU/mL [1]. The overall virus titer in each nasal wash sample was calculated as the mean titer of all valid dilutions of that sample.

## *Clinical symptom assessment*

- Total symptom score analysis: Participants were assessed for the severity of RSV challenge–related symptoms thrice per day, including during quarantine at the same time each day (±1 hour), using the hVIVO symptom diary card, collected on paper or electronically. The following 13-item symptom questionnaire was graded on a scale of 0–3 (grade 0: no symptoms; grade 1: just noticeable; grade 2: clearly bothersome from time to time but does not interfere with me doing my normal daily activities; grade 3: quite bothersome most or all of the time, and it stops me participating in activities); shortness of breath and wheeze had an additional grade, i.e., grade 4: symptoms at rest
  - Runny nose
  - Stuffy nose
  - Sneezing
  - Sore throat
  - Earache
  - Malaise/tiredness
  - Headache
  - Muscle and/or joint ache
  - Chilliness/feverishness
  - Cough
  - Chest tightness
  - Shortness of breath
  - Wheeze

## *Pharmacokinetic assay*

- Plasma samples of ~2 mL were collected for the measurement of plasma concentrations and calculation of pharmacokinetic parameters of S-337395, including maximum plasma concentration (C_max_), plasma concentration 24 hours post-dose and before subsequent dose (C_trough_), area under the curve for the concentration–time curve over the dosing interval (AUC_0-τ_), and terminal elimination half-life (t_1/2,z_).

# 5. Supplementary figures

## Supplementary Figure 1 Study design

Abbreviations: ITT-I, intent-to-treat infected; PI, principal investigator; qicPCR, qualitative integrative cycler polymerase chain reaction; RSV, respiratory syncytial virus.

^a^Screening assessments (including repeats, if required) were performed up to Day −1, at the discretion of the PI and per study design. Virus serology results were obtained within 90 days before challenge agent inoculation on Day 0.

^b^Discharge from quarantine was planned for Day 13 (13 days following inoculation) if the participant had no clinically significant symptoms. Virus detection by RSV discharge testing (eg, qualitative rapid viral antigen test) was performed at the discretion of the PI. If clinically significant symptoms and/or detectable virus persisted on Day 13, an extended quarantine stay could be required based on the PI’s assessment.

**Alt text:** Figure illustrates the study conduct design of this phase 2a trial.

## Supplementary Figure 2 Participant dissemination

Definitions of the analysis sets are as mentioned in Section 3 of the Supplementary Appendix

Abbreviations: AE, adverse event; ITT, intent-to-treat; ITT-A, intent-to-treat infected pre-dose; ITT-I, intent-to-treat infected; RSV+, infected with respiratory syncytial virus.

**Alt text:** Figure illustrates the randomized study population with different analysis sets based on onset of RSV infection.

# 6. Supplementary tables

## Supplementary Table 1 Demographic characteristics (ITT-A analysis set)

| Description | Placebo | S-337395 | | | | All |
| --- | --- | --- | --- | --- | --- | --- |
|  |  | **1 mg** | **10 mg** | **30 mg** | **300 mg** |  |
|  | **(n=15)** | **(n=5)** | **(n=10)** | **(n=10)** | **(n=9)** | **(N=49)** |
| Sex, n (%) |  |  |  |  |  |  |
| Female | 5 (33.3) | 2 (40.0) | 6 (60.0) | 2 (20.0) | 1 (11.1) | 16 (32.7) |
| Male | 10 (66.7) | 3 (60.0) | 4 (40.0) | 8 (80.0) | 8 (88.9) | 33 (67.3) |
| Age, years |  |  |  |  |  |  |
| Mean±SD | 25.47±6.47 | 32.40±8.53 | 26.20±5.22 | 31.20±7.90 | 26.33±7.33 | 27.65±7.16 |
| Median  (min, max) | 24.00  (18.0, 40.0) | 29.00  (25.0, 43.0) | 25.00  (19.0, 38.0) | 29.00  (21.0, 49.0) | 25.00  (20.0, 44.0) | 26.00  (18.0, 49.0) |
| Race, n (%) |  |  |  |  |  |  |
| Asian | 2 (13.3) | 0 (0.0) | 0 (0.0) | 2 (20.0) | 0 (0.0) | 4 (8.2) |
| Black or African American | 0 (0.0) | 0 (0.0) | 1 (10.0) | 0 (0.0) | 0 (0.0) | 1 (2.0) |
| White | 9 (60.0) | 5 (100.0) | 8 (80.0) | 7 (70.0) | 8 (88.9) | 37 (75.5) |
| Other | 1 (6.7) | 0 (0.0) | 1 (10.0) | 0 (0.0) | 1 (11.1) | 3 (6.1) |
| Multiple | 3 (20.0) | 0 (0.0) | 0 (0.0) | 1 (10.0) | 0 (0.0) | 4 (8.2) |
| Ethnicity, n (%) |  |  |  |  |  |  |
| Hispanic or Latino | 0 (0.0) | 0 (0.0) | 0 (0.0) | 0 (0.0) | 0 (0.0) | 0 (0.0) |
| Not Hispanic or Latino | 15 (100.0) | 5 (100.0) | 10 (100.0) | 10 (100.0) | 9 (100.0) | 49 (100.0) |

Percentages are based on the number of participants without missing values in the ITT-A analysis set.

Abbreviations: ITT-A, intent-to-treat infected pre-dose; max, maximum; min, minimum; SD, standard deviation.

## Supplementary Table 2 Efficacy analysis (ITT-A analysis set)

| Description | Placebo | S-337395 | | | |
| --- | --- | --- | --- | --- | --- |
|  |  | 1 mg | 10 mg | 30 mg | 300 mg |
|  | (n=15) | (n=5) | (n=10) | (n=10) | (n=9) |
| VL-AUC determined by qRT-PCR (hours×log_10_ copies/mL): primary efficacy analysis^a^ | | | | | |
| Mean±SD | 815.74±418.40 | 810.79±491.30 | 597.28±476.57 | 276.27±269.07 | 208.94±195.22 |
| Covariate-adjusted mean (95% CI) | 856.98 (684.93, 1029.03) | 739.41  (441.41, 1037.41) | 611.86  (402.50, 821.21) | 288.85  (79.52,  498.17) | 149.69  (−72.79,  372.18) |
| Difference in covariate-adjusted mean^b^ (95% CI) | - | −117.57 (−463.76, 228.62) | −245.13 (−515.57, 25.31) | −568.13 (−838.62, −297.64) | −707.29 (−990.66, −423.92) |
| *p*-value^c^ | - | 0.4971 | 0.0745 | 0.0001 | <0.0001 |
| Relative difference (%)^d^ | - | −13.72 | −28.60 | −66.29 | −82.53 |
| VL-AUC determined by viral culture (hours×log_10_ PFU/mL): secondary efficacy analysis^a^ | | | | | |
| Mean±SD | 304.40±183.16 | 314.70±289.87 | 220.27±206.53 | 77.52±105.44 | 30.64±30.62 |
| Covariate-adjusted mean (95% CI) | 303.42 (226.44, 380.41) | 328.45  (194.93, 461.96) | 202.93  (108.25, 297.61) | 78.37  (−15.91, 172.65) | 42.96  (−56.61,  142.53) |
| Difference in covariate-adjusted mean^b^ (95% CI) | - | 25.02  (−129.12, 179.16) | −100.49 (−222.49, 21.50) | −225.05 (−346.78, −103.33) | −260.47 (−386.35, −134.58) |
| *p*-value^c^ | - | 0.7450 | 0.1039 | 0.0006 | 0.0001 |
| Relative difference (%)^d^ | - | 8.25 | −33.12 | −74.17 | −85.84 |
| TSS-AUC from baseline up to Day 13 morning: secondary efficacy analysis (hours×score)^e^ | | | | | |
| Mean±SD | 416.04±411.59 | 340.91±297.49 | 357.51±474.59 | 185.43±173.88 | 107.09±146.69 |
| Covariate-adjusted mean (95% CI) | 469.83 (310.79, 628.86) | 354.64  (83.52,  625.75) | 323.18  (130.68, 515.67) | 151.10  (−41.39, 343.60) | 86.11  (−116.20, 288.42) |
| Difference in covariate-adjusted mean^b^ (95% CI) | - | −115.19 (−428.85, 198.47) | −146.65 (−398.40, 105.10) | −318.72 (−570.48, −66.97) | −383.72 (−642.28, −125.16) |
| *p*-value^c^ | - | 0.4630 | 0.2466 | 0.0143 | 0.0046 |
| Relative difference (%)^d^ |  | −24.52 | −31.21 | −67.84 | −81.67 |

Abbreviations: ANCOVA, analysis of covariance; CI, confidence interval; ITT-A, intent-to-treat infected pre-dose; PFU, plaque-forming unit; qRT-PCR, quantitative reverse-transcriptase polymerase chain reaction; SD, standard deviation; TSS, Total Symptom Score; TSS-AUC, area under the curve measurement for TSS; VL-AUC, viral load area under the curve.

^a^Baseline is the last non-missing observation before administration of the first dose of S-337395 or placebo.

^b^Comparison of each active dose with placebo.

^c^*p*-values are from ANCOVA, with treatment group as the main effect and baseline value as covariate.

^d^Relative difference is calculated as follows: (difference in covariate-adjusted mean for each active group/covariate-adjusted mean for placebo) ×100.

**^e^**Baseline is the closest non-missing observation to the administration of the first dose of study IMP. TSS is the sum of the following 10 symptoms: runny nose, stuffy nose, sneezing, sore throat, earache, malaise/tiredness, headache, muscle/joint ache, cough, and shortness of breath.

## Supplementary Table 3 Safety assessment and incidence of TEAEs by SOC and PT (safety analysis set)

| SOC  PT | Placebo | S-337395 | | | | All |
| --- | --- | --- | --- | --- | --- | --- |
|  |  | 1 mg | 10 mg | 30 mg | 300 mg |  |
|  | (n=26) | (n=10) | (n=26) | (n=26) | (n=26) | (N=114) |
| Any TEAEs | 2 (7.7) [2] | 4 (40.0) [5] | 6 (23.1) [7] | 10 (38.5) [10] | 6 (23.1) [6] | 28 (24.6) [30] |
| Infections  and infestations | 1 (3.8) [1] | 0 | 0 | 2 (7.7) [2] | 2 (7.7) [2] | 5 (4.4) [5] |
| Upper respiratory tract infection | 1 (3.8) [1] | 0 | 0 | 0 | 2 (7.7) [2] | 3 (2.6) [3] |
| Gastroenteritis viral | 0 | 0 | 0 | 1 (3.8) [1] | 0 | 1 (0.9) [1] |
| Urinary tract infection | 0 | 0 | 0 | 1 (3.8) [1] | 0 | 1 (0.9) [1] |
| Nervous system disorders | 0 | 1 (10.0) [1] | 2 (7.7) [2] | 2 (7.7) [2] | 0 | 5 (4.4) [5] |
| Dizziness | 0 | 0 | 1 (3.8) [1] | 1 (3.8) [1] | 0 | 2 (1.8) [2] |
| Paresthesia | 0 | 1 (10.0) [1] | 1 (3.8) [1] | 0 | 0 | 2 (1.8) [2] |
| Headache | 0 | 0 | 0 | 1 (3.8) [1] | 0 | 1 (0.9) [1] |
| Ear and labyrinth disorders | 1 (3.8) [1] | 0 | 0 | 0 | 0 | 1 (0.9) [1] |
| Ear pain | 1 (3.8) [1] | 0 | 0 | 0 | 0 | 1 (0.9) [1] |
| Respiratory, thoracic, and mediastinal disorders | 0 | 0 | 1 (3.8) [1] | 3 (11.5) [3] | 0 | 4 (3.5) [4] |
| Rhinorrhea | 0 | 0 | 1 (3.8) [1] | 1 (3.8) [1] | 0 | 2 (1.8) [2] |
| Cough | 0 | 0 | 0 | 1 (3.8) [1] | 0 | 1 (0.9) [1] |
| Nasal congestion | 0 | 0 | 0 | 1 (3.8) [1] | 0 | 1 (0.9) [1] |
| Skin and subcutaneous tissue disorders | 0 | 0 | 1 (3.8) [1] | 0 | 2 (7.7) [2] | 3 (2.6) [3] |
| Dermatitis | 0 | 0 | 0 | 0 | 1 (3.8) [1] | 1 (0.9) [1] |
| Dry skin | 0 | 0 | 1 (3.8) [1] | 0 | 0 | 1 (0.9) [1] |
| Rash | 0 | 0 | 0 | 0 | 1 (3.8) [1] | 1 (0.9) [1] |
| Musculoskeletal and connective tissue disorders | 0 | 0 | 1 (3.8) [1] | 2 (7.7) [2] | 1 (3.8) [1] | 4 (3.5) [4] |
| Musculoskeletal chest pain | 0 | 0 | 0 | 0 | 1 (3.8) [1] | 1 (0.9) [1] |
| Myalgia | 0 | 0 | 0 | 1 (3.8) [1] | 0 | 1 (0.9) [1] |
| Pain in jaw | 0 | 0 | 1 (3.8) [1] | 0 | 0 | 1 (0.9) [1] |
| Temporomandibular pain and dysfunction syndrome | 0 | 0 | 0 | 1 (3.8) [1] | 0 | 1 (0.9) [1] |
| General disorders and administration site conditions | 0 | 2 (20.0) [2] | 0 | 0 | 0 | 2 (1.8) [2] |
| Chest discomfort | 0 | 1 (10.0) [1] | 0 | 0 | 0 | 1 (0.9) [1] |
| Chest pain | 0 | 1 (10.0) [1] | 0 | 0 | 0 | 1 (0.9) [1] |
| Investigations | 0 | 2 (20.0) [2] | 1 (3.8) [1] | 1 (3.8) [1] | 1 (3.8) [1] | 5 (4.4) [5] |
| Alanine aminotransferase increased | 0 | 1 (10.0) [1] | 0 | 0 | 1 (3.8) [1] | 2 (1.8) [2] |
| Neutrophil count decreased | 0 | 1 (10.0) [1] | 1 (3.8) [1] | 0 | 0 | 2 (1.8) [2] |
| Electrocardiogram T-wave abnormal | 0 | 0 | 0 | 1 (3.8) [1] | 0 | 1 (0.9) [1] |
| Injury, poisoning, and procedural complications | 0 | 0 | 1 (3.8) [1] | 0 | 0 | 1 (0.9) [1] |
| Limb injury | 0 | 0 | 1 (3.8) [1] | 0 | 0 | 1 (0.9) [1] |

Note: Outcomes are presented as the number of participants (percentage) [number of events].

Coded using MedDRA version 27.0. Abbreviations: MedDRA, Medical Dictionary for Regulatory Activities; PT, Preferred Term; SOC, System Organ Class; TEAE, treatment-emergent adverse event.

## Supplementary Table 4 Pharmacokinetic parameters after multiple doses of S-337395 (pharmacokinetic analysis set)

|  | Plasma S-337395 | | | | | | | |
| --- | --- | --- | --- | --- | --- | --- | --- | --- |
|  | **1 mg QD** | | **10 mg QD** | | **30 mg QD** | | **300 mg QD** | |
| Parameters | **Day 1** | **Day 5** | **Day 1** | **Day 5** | **Day 1** | **Day 5** | **Day 1** | **Day 5** |
| n | **10** | **10** | **26** | **26** | **26** | **25** | **26** | **26** |
| C_max_ (ng/mL) | 14.1  (29.1) | 22.4  (29.6) | 135  (35.7) | 197  (49.1) | 336  (36.7) | 561  (38.7) | 1610  (43.9) | 2620  (39.6) |
| C_trough_ (ng/mL) | 5.42  (30.2) | 8.95  (47.8) | 43.5  (45.8) | 67.6  (66.7) | 111  (48.7) | 208  (62.7) | 502  (51.7) | 972  (61.8) |
| T_max_ (hours)^a^ | 6.03 (3.90, 13.37) | 6.86 (2.47, 11.95) | 4.96 (2.42, 12.27) | 3.94 (1.95, 12.00) | 4.96 (2.43, 11.92) | 2.95 (2.08, 6.03) | 2.90 (1.45, 8.07) | 2.96 (0.95, 4.00) |
| AUC_0-τ_ (ng.hours/mL) | 214.9  (22.0) | 362.2  (32.8) | 1972 (35.0) | 3019 (52.8) | 4997 (35.8) | 8877 (44.2) | 22560 (39.5) | 40710 (43.5) |
| t_1/2,z_ (hours) | NC | 16.9  (28.5) | NC | 16.1 (27.4) | NC | 16.3 (26.8) | NC | 16.8  (30.0) |

Data are presented as geometric mean (%CV geometric mean).

Abbreviations: AUC_0-τ_, area under the plasma concentration–time curve over the dosing interval; C_max_, maximum plasma concentration; C_trough_, plasma concentration 24 hours post-dose and before subsequent dose; CV, coefficient of variation; max, maximum; min, minimum; NC, not calculated; QD, once daily; T_max_, time to C_max_; t_1/2,z_, terminal elimination half-life.

^a^T_max_ (hours) data are presented as median (min, max).

# 7. References

1. Cheng MH, Mann AJ, Maas BM, et al. A phase 2a, randomized, placebo-controlled human challenge trial to evaluate the efficacy and safety of molnupiravir in healthy participants inoculated with respiratory syncytial virus. Pulm Ther, 2025;11:285-304.
2. Kelly G, Laxton C, Garelnabi M, et al. Use of qualitative integrative cycler PCR (qicPCR) to identify optimal therapeutic dosing time-points in a respiratory syncytial virus human viral challenge model (hVCM). J Virol Methods, 2015;224:83-90.
